# Cerebral “Dirty-Appearing” White Matter in Relation to Cognitive Decline and Dementia Risk in Community-Dwelling Older Adults

**DOI:** 10.1212/WNL.0000000000218036

**Published:** 2026-05-08

**Authors:** Ingmar Eiling, Sigurdur Sigurdsson, Jasmin Annica Kuhn-Keller, Simon P. Mooijaart, Lenore J. Launer, Matthias J.P. Van Osch, Vilmundur Gudnason, Jeroen de Bresser

**Affiliations:** 1 Department of Radiology, Leiden University Medical Center, the Netherlands;; 2 Icelandic Heart Association, Kopavogur, Iceland;; 3 Department of Gerontology and Geriatrics, LUMC, Leiden, the Netherlands;; 4 LUMC Center for Medicine for Older People, LUMC, Leiden, the Netherlands;; 5 Laboratory of Epidemiology and Population Science, National Institute on Aging, Bethesda, MD; and; 6 Faculty of Medicine, University of Iceland, Reykjavik.

## Abstract

**Background and Objectives:**

Cerebral “dirty-appearing” or “diffusely abnormal” white matter (DAWM) represents subtle white matter abnormalities that are associated with progression of cerebral small vessel disease (cSVD), but the association with cognitive function and dementia is unclear. Our aim was to study the association between DAWM and cognitive function, long-term cognitive decline, and long-term dementia risk in community-dwelling older adults with limited baseline cSVD burden.

**Methods:**

Participants of the prospective Age-Gene/Environment Susceptibility–Reykjavik longitudinal cohort study underwent 1.5T brain MRI scans. DAWM was visually rated on baseline fluid-attenuated inversion recovery (FLAIR) MRI as a percentage of lobar white matter volume (0%, 0%–10%, 10%–25%, or >25% DAWM) per brain lobe. Baseline DAWM ratings and white matter hyperintensity (WMH) volumes were associated with memory, executive function, and processing speed cognitive domain *z*-scores at baseline and their change at follow-up (after 5.2 ± 0.2 years) and with dementia status assessed at long-term follow-up (after 10.3 ± 2.2 years). This was performed by statistical models adjusted for age, sex, vascular risk factors, and education (for the cognition analyses).

**Results:**

From 4,163 included participants, 2,081 participants were selected based on limited baseline WMH volume on FLAIR MRI (determined by a median split) (mean age: 74.6 ± 4.9 years, 61% female). Baseline DAWM ratings were not associated with baseline cognition *z*-scores (memory: *B* = 0.01 [−0.03 to 0.04], *p* = 0.637; executive function: *B* = 0.02 [−0.05 to 0.01], *p* = 0.244; processing speed: *B* = −0.01 [−0.04 to 0.01], *p* = 0.383), nor with cognitive decline after 5 years (memory: *B* = 0.03 [−0.01 to 0.06], *p* = 0.119); executive function: *B* = 0.01 [−0.02 to 0.04], *p* = 0.508; processing speed: *B* = −0.01 [−0.03 to 0.02], *p* = 0.498), nor with increased risk of dementia after 10 years (hazard ratio [HR] 0.93 [0.84–1.03], *p* = 0.156). By contrast, baseline WMH volume was associated with baseline cognition *z*-scores (executive function: *B* = −0.09 [−0.16 to −0.02], *p* = 0.011; processing speed: *B* = −0.08 [−0.15 to −0.02], *p* = 0.007), cognitive decline in processing speed after 5 years (*B* = −0.06 [−0.12 to −0.01], *p* = 0.024), and with a higher dementia risk after 10 years (HR 1.35 [1.04–1.73], *p* = 0.025).

**Discussion:**

In contrast to WMH, DAWM was not associated with baseline cognition, long-term cognitive decline, nor long-term dementia risk in community-dwelling older adults with limited cSVD burden. Although DAWM is associated with progression of cSVD, its role in the development of cognitive impairment and dementia remains unclear.

## Introduction

Cerebral small vessel disease (cSVD) in older adults is a major contributor to cognitive decline and dementia, also in adults with Alzheimer disease.^1-4^ Early cSVD markers are urgently needed to allow for selection of individuals for preventative treatment such as lifestyle interventions or for future treatment trials aimed at prevention of cognitive decline and dementia.

The most common marker of cSVD on brain MRI is the presence of white matter hyperintensities (WMHs) of presumed vascular origin.^5^ On a group level, WMH volume is associated with long-term cognitive decline^6^ and dementia risk^7^ in community-dwelling older adults. However, there is considerable variation in the strength of these associations between studies and on an individual level. Furthermore, large WMH can be considered late brain changes of cSVD.^6,7^ Investigating individuals with limited WMH burden and more subtle white matter changes might help to better characterize the earlier stages of cSVD and its relation to dementia and cognitive decline.^8^

Improvements in clinical MRI (such as higher contrast and resolution) have allowed for the visualization of more subtle white matter changes, especially on T2/fluid-attenuated inversion recovery (FLAIR) MRI scans.^9,10^ These changes can occur alongside or separate from WMH and are called “dirty-appearing” or “diffusely abnormal” white matter (DAWM).^10^ In a previous study on DAWM in community-dwelling older adults, using a previously developed visual rating scale,^11^ we have defined DAWM as areas of diffuse T2/FLAIR signal hyperintensity in between normal-appearing white matter and WMH.^9^ In that previous study, a higher DAWM burden was associated with cSVD progression after 5 years (i.e., increased WMH volumes and increased risk of subcortical infarcts). DAWM might therefore be an early marker of cSVD and might precede development of WMH.

It is currently unknown if DAWM is associated with cognitive decline and dementia outcomes. Therefore, our aim was to study the association between DAWM and cognitive function, long-term cognitive decline, and long-term dementia risk in community-dwelling older adults with limited baseline cSVD burden.

## Methods

### Study Population and Design

Community-dwelling older adults were recruited from the Age-Gene/Environment Susceptibility (AGES) Reykjavik study, a prospective longitudinal cohort study.^12^ Individuals in this cohort were born between 1907 and 1935. A total of 4,614 participants underwent 1.5T brain MRI scans and cognitive testing procedures at baseline from 2002 onwards in Reykjavik, Iceland. This study performs a retrospective analysis on this cohort.

### Standard Protocol Approvals, Registrations, and Patient Consents

All participants signed written informed consent before participating in the baseline and follow-up study. The study was approved by the Institutional Review Board of Iceland (IRB, VSN 00-063), the Icelandic Data Protection Committee, and the IRB serving the National Institute on Aging, in accordance with the Declaration of Helsinki.^12^

### Procedures

Baseline dementia status was assessed as follows: participants with a score <24 on the Mini-Mental State Examination or <18 on the Digit Symbol Substitution Test (DSST),^13^ coupled with poor subsequent performance on the Auditory Verbal Learning Test^14^ or the Trail Making Test part B, were administered a full neurologic status assessment and medical anamnesis. Further diagnostic information included a clinical brain MRI, hearing and vision tests, a blood panel including vitamin B12 and thyroid hormones, and social and functional activities of daily living assessment. Follow-up dementia diagnoses were tracked through study visits, vital statistics, hospital records, and the Resident Assessment Instrument^15^ at home or in nursing homes, which describes health status and disability. Based on all available information and following international guidelines,^16^ a multidisciplinary panel (neurologist, geriatrician, neuropsychologist, and neuroradiologist in case of clinical MRI reading) reached a consensus on an outcome of either dementia, mild cognitive impairment, or normal cognitive function.^12^

For baseline and follow-up cognitive assessment, a cognitive test battery was used comprised of the California Verbal Learning Test,^17^ Digits Forward and Backwards,^13^ Figure Comparison,^18^ DSST,^13,18^ and a Stroop Test. Test results were *z*-scored within 3 cognitive domains: memory, executive function, and processing speed.^19^ Cognitive decline per domain was calculated by subtracting the baseline measurement *z*-score from a follow-up measurement collected 5.2 ± 0.2 (SD) years later. This resulted in a negative score if a follow-up score was lower than the individual's baseline scores and a positive score if a follow-up score was higher.

Participant information was collected using questionnaires. Their highest education level was recorded as primary school, secondary school, college, or university. Former tobacco smokers were defined as having smoked at least 100 cigarettes or 20 cigars, nonsmokers smoked less than that or have never smoked, and current smokers were smoking actively at baseline. The body mass index (BMI) was calculated from self-reported height and weight. Systolic and diastolic blood pressure were averaged over 2 measurements, using a mercury sphygmomanometer. Hypertension status was defined by antihypertensive medication use, or by >140 mm Hg systolic and/or >90 mm diastolic blood pressure, or self-report. Diabetes mellitus status was defined by antidiabetic medication use, fasting blood glucose >7 mmol/L, or self-report. Coronary artery disease was defined by angina plus nitrate use, electrocardiogram evidence of myocardial infarction, or self-report.

### MRI Acquisition Protocol

MR images were acquired on a 1.5 Tesla Signa Twinspeed EXCITE (GE, Milwaukee, WI) with a 4-channel or 8-channel phased array head coil. Sequences included a 3D T1-weighted spoiled gradient echo (echo time [TE]/repetition time [TR] = 8/21 milliseconds, flip angle [FA] 30°, 240 mm field of view [FOV], 0.94 × 0.94 × 1.5 mm^3^ voxels), T2-weighted FLAIR (TE/TR/inversion time [TI] = 100/8,000/2,000 milliseconds, no fat suppression), T2*-weighted gradient echo echo-planar imaging (TE/TR = 50/3,050 milliseconds), and a proton density/T2-weighted fast spin-echo (TE1/TE2/TR = 22/90/3,220 milliseconds, echo train length = 8). The latter 3 sequences had a 256 × 256 acquisition matrix with 220 mm FOV, 90° FA, 0.86 × 0.86 × 3 mm^3^ voxels with interleaved slices, and were angulated to the anterior-posterior commissural line.

### Cerebral Small Vessel Disease MRI Markers

White matter and WMH volumes were segmented from baseline MRI scans using a modified algorithm based on the Montreal Neurological Institute (MNI) pipeline.^20^ Cerebral subcortical infarcts, enlarged perivascular spaces (ePVS), and microbleeds were identified visually by neuroradiologists and subsequently by 2 trained radiographers with excellent interrater agreement.^21,22^ Subcortical infarcts were defined as parenchymal defects with central signals isointense to CSF across all acquired MRI sequences, not extending to the cortex, and surrounded by a FLAIR-hyperintense area measuring at least 4 mm in diameter. Microbleeds were classified as focal signal voids on T2*-weighted images that were smaller or invisible on T2-weighted images.^21^ ePVS were defined as tubular signal changes lacking a FLAIR-hyperintense area and hemosiderin deposition on T2*-weighted imaging. They were differentiated from subcortical infarcts by their often smaller size and absence of adjacent FLAIR hyperintensity.

### Dirty-Appearing White Matter Rating

Before rating, T1-weighted images were registered to 2009c MNI space (affine registration; B-spline interpolation) using ITK-Elastix^23^ (0.10) in Python 3.8.5 (Python Software Foundation, Wilmington, DE). FLAIR images were coregistered to T1-weighted and transformed to MNI space using the T1-weighted affine matrix. No registration distortions were found in the registered FLAIR images.

**Figure 1:**
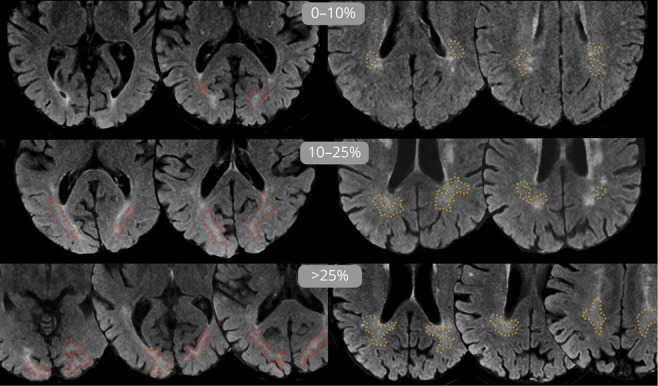
Examples of DAWM on FLAIR MRI Slices From 6 Individuals Transversal slices of 1.5T FLAIR MRI scans of 6 different older adult participants after skull removal by image processing. DAWM in the occipital white matter is delineated by red dotted lines, and DAWM in the parietal white matter is delineated by orange dotted lines. Dotted lines are for illustration purposes only because DAWM is, by definition, difficult to precisely delineate (and was therefore visually rated instead). Note the difference in apparent DAWM volume between the top row (<10% DAWM of lobar white matter), where only mostly small WMH and ependymal lining is visible, and the second and third rows (10%–25% and >25% of lobar white matter), where larger, more confluent configurations of DAWM can be seen, extending beyond the slices shown here. DAWM = dirty-appearing white matter; FLAIR = fluid-attenuated inversion recovery; WMH = white matter hyperintensity.

The volume of DAWM was estimated relative to the volume of the frontal, temporal, parietal, and occipital white matter, defined through landmarks.^9^ This was performed by using a visual rating scale^11^ (0: no DAWM, 1: >0%–10% of lobar white matter, 2: >10%–25% of lobar white matter, 3: >25% of lobar white matter; Figure 1), and these lobar ratings were summed per individual.

DAWM was defined as an area of diffuse, subtle T2/FLAIR signal intensity with a minimal axial diameter of 3 mm in between normal-appearing white matter and WMH, visible on at least 2 axial slices, and with at least a 3 mm distance from WMH edges in the axial and sagittal planes. As the slice thickness was 3 mm, these criteria prevented the rating of DAWM in areas with partial volume effects of WMH edges that would present with lower T2 intensity compared with the center of WMH. DAWM was distinguished from the fanning fibers of the thalamocortical projections, optic radiation, and corona radiata by anatomical location and selection on an irregular shape with a soft edge. DAWM was not rated adjacent to infarcts. Scans of participants with very large infarcts that occupied most of the white matter were excluded because this prohibited rating of DAWM, but this finding was rare (n = 26). DAWM could be distinguished from ePVS because these were generally not T2/FLAIR hyperintense on 1.5T FLAIR MRI, but care was taken to avoid tubular hyperintense structures on regular T2-weighted scans or with suboptimal FLAIR fluid suppression. Window and level settings were optimized for DAWM rating in all participants by increasing contrast and tuning the brightness, resulting in nearly black basal ganglia and dark-gray normal-appearing white matter. Criteria were defined in collaboration with an experienced neuroradiologist (J.d.B.), and consensus meetings were regularly performed. The rating criteria resulted in participants with large confluent WMH rarely showing DAWM due to our safety margin around WMH and the WMH taking up most of the white matter in which DAWM could occur. Two raters performed the DAWM rating while blinded to any patient characteristics, with good to excellent intrarater and interrater reliability (linear weighted κ = 0.72–0.91).^9^

### Statistical Analysis

The associations between baseline DAWM and baseline cognition as well as cognitive decline per domain were analyzed with linear regression models. Statistical assumptions were checked using histograms and Q-Q plots. A first statistical model included baseline age, sex, and education level. A second model additionally adjusted for vascular risk factors (hypertension, type 2 diabetes mellitus, smoking status, and BMI). As a reference for the DAWM analyses, these 2 analyses were also performed with log-corrected baseline WMH volume with white matter volume as additional covariate. As sensitivity analyses, the association between baseline DAWM and baseline cognition was retested by including the participants with missing follow-up cognition data (eAppendix 1).

The association between baseline DAWM and dementia outcomes was analyzed by 2 Cox proportional hazards models; the first model was adjusted for baseline age, sex, and education level, and the second model was additionally adjusted for vascular risk factors (hypertension, type 2 diabetes mellitus, smoking status, and BMI). As a reference for the DAWM analyses, these analyses were also performed with natural log-corrected baseline WMH volume and additionally adjusted for white matter volume.

Sensitivity analyses were performed to better assess the complex relation between DAWM and WMH burden on dementia outcomes using Cox proportional hazard models. First, baseline DAWM was related to dementia outcomes in the full sample while adjusting for WMH and white matter volumes alongside age, sex, and vascular risk factors (eAppendix 2). Furthermore, the association of DAWM and WMH with dementia outcome was analyzed in a combined way in a large subsample (n = 2,449) using DAWM pseudovolumes calculated from white matter volumes multiplied by weights derived from the DAWM ratings per lobe. Model performance was compared for these pseudovolumes in 2 separate models with a reference model with only WMH volumes (eAppendix 3 and eTable 1).

In all Cox proportional hazard models, a global Schoenfeld test with a *p* value higher than 0.05 suggested that the proportional hazards assumption was met. Data preparation and analysis were performed in R Studio (R 4.3.3).

### Data Availability

The AGES I-II data set cannot be made publicly available because the informed consent signed by the participants prohibits public data sharing. This was outlined by the study approval by the Icelandic National Bioethics Committee. Data requests may be sent to the AGES-Reykjavik Study Executive Committee, contact: Vilmundur Gudnason, v.gudnason@hjarta.is.

## Results

**Figure 2:**
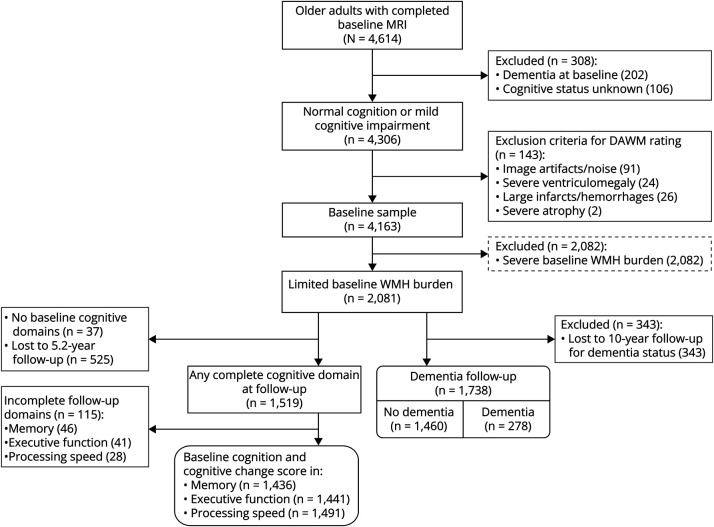
Flowchart Describing Exclusion Criteria for DAWM Rating and Analysis Steps Subgroups were selected for the 3 main analysis outcomes (baseline cognition, cognitive decline, and dementia risk) from the total group of participants with limited baseline WMH burden (n = 2,081). For baseline cognition and cognitive decline, each domain was assessed independently and did not depend on data availability of other complete domains. DAWM = dirty-appearing white matter; WMH = white matter hyperintensity.

**Figure 3:**
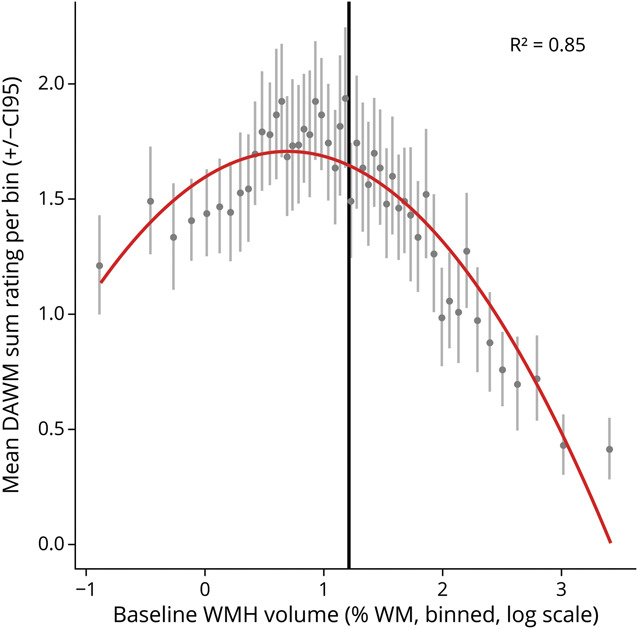
Relation Between Binned Baseline DAWM Sum Ratings and Baseline WMH Volume This figure illustrates the nonlinear relationship between baseline DAWM ratings and baseline WMH volume. It shows the mean DAWM sum rating for 50 bins ranging the entire distribution of baseline WMH volumes (normalized for white matter volume, total sample: n = 4,163, bin size: n = 83). The bins are centered for their mean WMH volume on a log-scaled x-axis. A quadratic fit (red line) describes this nonlinear relationship well, as summarized by a high *R*^2^ of the fit on the centered bin means. The log scaling helps to emphasize the inverse U-shape of this quadratic fit. The vertical line shows the point of median split (3.36% WMH volume of total baseline white matter volume). DAWM = dirty-appearing white matter; WMH = white matter hyperintensity.

Participants were excluded from our study if they had dementia (n = 202) or an unknown cognitive status (n = 106) at baseline, if their baseline MRI scan contained high noise or artefacts such as strong Gibbs ringing (n = 91), or if extensive parts of the white matter showed pathologic changes such as infarcts (n = 28). We did not exclude cases of minor white matter atrophy or minor ventriculomegaly. Our included baseline sample then consisted of 4,163 participants from whom we included individuals with limited baseline WMH burden (n = 2,081) for further analysis (Figure 2). These participants were included based on a median split on WMH volume normalized for white matter volume. In a previous study,^9^ this approach was performed to focus on individuals with relatively limited WMH burden because high WMH volumes preclude DAWM from being observed in the same white matter areas. Furthermore, it provides a natural cutoff to focus the analysis on individuals with relatively high DAWM scores and relatively low WMH volumes (Figure 3).

Baseline characteristics of the community-dwelling older adults with limited baseline WMH burden (n = 2,081) are summarized in Table 1. These individuals with limited baseline WMH burden have more DAWM on average compared with individuals with severe WMH burden (Figure 3). The community-dwelling older adults with limited baseline WMH burden were on average 74.6 ± 4.9 years old at baseline, and 61% were female. These participants were assessed for baseline cognition, cognitive decline after 5.2 ± 0.2 years, and dementia outcomes after 10.3 ± 2.2 years (median: 10.7 years).

**Table 1:** Baseline Characteristics of the Study Group

Baseline characteristic	Older adults with limited baseline WMH burden (n = 2,081)
Age (y)	74.6 ± 4.9
Female	1,259 (61%)
Education	
Primary school	359 (17%)
Secondary school	886 (43%)
College	293 (14%)
University	195 (9%)
BMI (kg/m^2^)	27.1 ± 4.3
Hypertension	1,575 (76%)
Coronary artery disease	300 (14%)
Diabetes mellitus type 2	192 (9%)
Tobacco use	
Never	963 (46%)
Former	918 (44%)
Current	200 (10%)
cSVD markers	
DAWM sum rating	1.67 ± 1.15
WMH volume (mL)	7.1 (4.8–9.8%)
Subcortical infarcts	85 (5%)
Microbleeds	184 (11%)
ePVS	231 (13%)

Abbreviations: AGES = Age-Gene/Environment Susceptibility; BMI = body mass index; cSVD = cerebral small vessel disease; DAWM = dirty-appearing white matter; ePVS = enlarged perivascular spaces; WMH = white matter hyperintensity.

Values are given as mean ± SD or n (%) except for WMH volume, which was given as a median and interquartile range. BMI is shown in kilograms per meter squared. Subcortical infarcts, microbleeds, and ePVS were defined as having any number of them at baseline (baseline AGES-Reykjavik cohort^22^).

### DAWM and WMH in Relation to Baseline Cognitive Function

In community-dwelling older adults with limited baseline WMH burden, DAWM sum ratings were not significantly associated with baseline cognition *z*-scores in the memory domain (*B* = 0.01 [−0.03 to 0.04], *p* = 0.637), nor in the executive function domain (*B* = −0.02 [−0.05 to 0.01], *p* = 0.244), or the processing speed domain (*B* = −0.01 [−0.04 to 0.01], *p* = 0.383) when adjusting for age, sex, and vascular risk factors (model 2, Table 2). Not adjusting for vascular risk factors showed similar results (model 1, Table 2). Sensitivity analyses including participants with missing follow-up cognition data also showed similar results (eAppendix 1). Reference analyses (Table 2) showed that a higher baseline WMH volume was significantly associated with lower baseline cognition *z*-scores in the executive function domain (*B* = −0.09 [−0.16 to −0.02], *p* = 0.011) and in the processing speed domain (*B* = −0.08 [−0.15 to −0.02], *p* = 0.007), but not in the memory domain (*B* = −0.07 [−0.15 to 0.01], *p* = 0.104).

**Table 2:** Baseline DAWM and WMH in Relation to Baseline Cognitive Function

Domains at baseline	Baseline *z*-score Mean ± SD	DAWM model 1 *B* (95% CI)	DAWM model 2 *B* (95% CI)	WMH model 1 *B* (95% CI)	WMH model 2 *B* (95% CI)
Memory (n = 1,436)	0.18 ± 0.90	0.01 (−0.03 to 0.04)	0.01 (−0.03 to 0.04)	−0.07 (−0.15 to 0.01)	−0.07 (−0.15 to 0.01)
Executive function (n = 1,441)	0.12 ± 0.74	−0.02 (−0.05 to 0.01)	−0.02 (−0.05 to 0.01)	−0.09^a^ (−0.16 to −0.02)	−0.09^a^ (−0.16 to −0.03)
Processing speed (n = 1,491)	0.16 ± 0.72	−0.01 (−0.04 to 0.01)	−0.01 (−0.04 to 0.02)	−0.09^a^ (−0.15 to −0.03)	−0.08^a^ (−0.15 to −0.03)

Abbreviations: DAWM = dirty-appearing white matter; WMH = white matter hyperintensity.

For the values shown for the DAWM analyses (DAWM models 1 and 2), linear regressions were performed to associate baseline DAWM with cognitive domain *z*-score at baseline, resulting in *B* values and 95% CIs. For the reference analyses (WMH models 1 and 2), baseline normalized and natural log-corrected WMH volume was associated with cognitive domain *z*-scores at baseline. Model 1 was adjusted for the covariates age, sex, and baseline education level, while model 2 was additionally adjusted for vascular risk factors. Both WMH models were additionally adjusted for white matter volume.

a *p* < 0.05.

### DAWM and WMH in Relation to Cognitive Decline After 5 Years

In community-dwelling older adults with limited baseline WMH burden, DAWM sum ratings were not significantly associated with cognitive decline at 5-year follow-up in the memory domain (*B* = 0.03 [−0.01 to 0.06], *p* = 0.119), nor in the executive function domain (*B* = 0.01 [−0.02 to 0.04], *p* = 0.508), or processing speed domain (*B* = −0.01 [−0.03 to 0.02], *p* = 0.498) when adjusting for age, sex, and vascular risk factors (model 2, Table 3). Not adjusting for vascular risk factors showed similar results (model 1, Table 3). Reference analyses showed that a higher baseline WMH volume was significantly associated with increased cognitive decline in the processing speed domain (*B* = −0.06 [−0.12 to −0.01], *p* = 0.024), but showed no significant association for the memory domain (*B* = −0.07 [−0.14 to 0.00], *p* = 0.058) or the executive function domain (*B* = 0.03 [−0.04 to 0.10], *p* = 0.328) (Table 3).

**Table 3:** Baseline DAWM and WMH in Relation to Cognitive Decline After 5 Years

Domains at 5-y follow-up	*z*-score change Mean ± SD	DAWM model 1 *B* (95% CI)	DAWM model 2 *B* (95% CI)	WMH model 1 *B* (95% CI)	WMH model 2 *B* (95% CI)
Memory (n = 1,436)	−0.19 ± 0.71	0.03 (−0.01 to 0.06)	0.03 (−0.01 to 0.06)	−0.07 (−0.14 to 0.00)	−0.07 (−0.14 to 0.00)
Executive function (n = 1,441)	−0.21 ± 0.67	0.01 (−0.02 to 0.04)	0.01 (−0.02 to 0.04)	0.03 (−0.04 to 0.10)	0.03 (−0.03 to 0.10)
Processing speed (n = 1,491)	−0.28 ± 0.57	−0.01 (−0.03 to 0.02)	−0.01 (−0.03 to 0.02)	−0.06^a^ (−0.12 to −0.01)	−0.06^a^ (−0.12 to −0.01)

Abbreviations: DAWM = dirty-appearing white matter; WMH = white matter hyperintensity.

For the values shown under DAWM analyses (DAWM models 1 and 2), linear regressions with *B* values and 95% CIs are shown for the association of baseline DAWM with cognitive decline from baseline to follow-up. For the reference analyses (WMH models 1 and 2), linear regressions were performed to associate baseline normalized and natural log-corrected WMH volume with cognitive decline from baseline to follow-up. Model 1 was adjusted for the covariates age, sex, and baseline education level, while model 2 was additionally adjusted for vascular risk factors. Both WMH models were additionally adjusted for white matter volume.

a *p* < 0.05.

### DAWM and WMH in Relation to Dementia Risk After 10 Years

In community-dwelling older adults with limited baseline WMH burden, dementia status was determined 10.3 ± 2.2 years (median: 10.7 years) after baseline when participants were on average 84.6 ± 4.2 years of age. Compared with participants who did not develop dementia, participants who developed dementia were on average 3.9 years older at baseline, had lower education levels, had a higher BMI, and were more often former smokers. A total of 278 participants of 1,738 (16%) were diagnosed with dementia at follow-up (eTable 2).

In the older adults with limited baseline WMH burden, baseline DAWM sum rating was not significantly associated with dementia risk after 10 years (hazard ratio [HR] 0.93 [95% CI 0.84–1.03], *p* = 0.156). Not adjusting for vascular risk factors showed comparable results (HR 0.93 [0.84–1.03], *p* = 0.157). By contrast, the reference analysis showed that a higher baseline WMH volume was associated with higher dementia risk after 10 years (HR 1.35 [1.05–1.75], *p* = 0.025). Again, not adjusting for vascular risk factors showed comparable results (HR 1.36 [1.05–1.75], *p* = 0.020).

Sensitivity analyses were performed to better assess the complex relation between DAWM and WMH burden regarding dementia outcomes (eAppendix 2). In available participants from the full cohort with limited and severe WMH burden (n = 3,377), higher baseline DAWM ratings were associated with a reduced long-term risk of dementia even when adjusting for WMH, white matter volumes, and other covariates. The effect was only attenuated when using an interaction term between DAWM sum rating and WMH volume. Therefore, because of the inverted U-shape association between DAWM and WMH in the full sample, more complex statistical models are needed to not mistakenly suggest that DAWM is associated with a lower dementia risk. Other sensitivity analyses were performed in participants with lobar white matter volume segmentations available (n = 2,449) (eAppendix 3). These analyses showed that total abnormal white matter volume (calculated by adding DAWM pseudovolumes to WMH volumes) slightly increases the hazard ratio to estimate dementia risk with compared with a model with WMH volumes only.

## Discussion

In community-dwelling older adults with limited baseline cSVD burden, DAWM sum ratings were not associated with baseline cognition nor cognitive decline after 5 years in the memory, executive function, or processing speed domains. DAWM sum ratings were also not associated with increased risk of dementia after 10 years. By contrast, baseline WMH volume was associated with baseline cognition in the executive function and processing speed domains, cognitive decline after 5 years in the processing speed domain, and higher dementia risk after 10 years.

The presence of extensive WMH can be viewed as a late manifestation of cSVD, whereas DAWM may reflect earlier changes. In our previous study,^9^ baseline DAWM was associated with increased WMH progression and higher odds of new subcortical infarcts after 5 years in community-dwelling older adults with limited baseline cSVD burden. This study is the first to examine DAWM in relation to long-term cognitive decline and dementia in community-dwelling older adults with limited cSVD burden. However, in our study, we did not find an association of baseline DAWM with baseline cognition, cognitive decline at 5 years, nor increased dementia risk at 10 years. These findings show the need to reassess which MRI markers of subtle white matter changes best associate with long-term cognitive outcomes and dementia, especially in individuals with limited cSVD burden.

Several other approaches have been developed to study subtle white matter changes on MRI, using a combination of FLAIR and diffusion tensor imaging. One study in older adults with and without cognitive impairment (n = 119) divided the normal-appearing white matter into subregions based on FLAIR intensity *z*-scores.^24^ They showed that regions with higher FLAIR intensity and lower fractional anisotropy contained more severe WMH increases over a follow-up period. Other studies have instead focused on diffusivity changes located directly adjacent to WMH; the so-called WMH penumbra.^25^ In older patients without dementia, elevated diffusivity in the WMH penumbra was linked to the progression of WMH volumes at follow-up.^26^ It is important that in the context of cognition, reduced white matter integrity in the WMH penumbra was linked to lower cognitive screener scores in patients with cerebrovascular disease (n = 58),^27^ but this was not found using more comprehensive cognitive assessments in patients with cSVD and mild cognitive impairment (n = 73).^28^ We are not aware of studies associating the WMH penumbra or related concepts with long-term cognition or dementia outcome, which suggests that our study provides scarce longitudinal evidence on the lack of an association between subtle white matter changes and long-term cognitive decline and dementia risk.

Total WMH volume in older adults is associated with cognitive function and cognitive decline, especially in the executive function and memory cognitive domains, and this association is stronger than for other cSVD markers.^6^ This study on older adults with limited baseline cSVD burden showed that a higher baseline WMH volume was associated with lower baseline processing speed and executive function. We also showed an association with faster decline in processing speed after 5 years. Such declines in processing speed have been linked to reduced local white matter network efficiency.^29^ By contrast, in our study, baseline WMH volume was not related to memory function or its 5-year decline, which suggests that the natural variability of memory scores can be quite high compared with other community-dwelling cohorts.^6^ In a recent study on older adults, an annual −0.30 to −0.37 *z*-score change within a cognitive domain, which amounts to an approximate −1.5 *z*-score change over 5 years, was linked to conversion from normal cognition to MCI.^30^ This *z*-score change could be suggested as a threshold to define cognitive decline as clinically meaningful. Only a very small proportion of our participants met this definition of clinically meaningful cognitive decline, which implies that most of the observed cognitive decline was subclinical.

Population-based studies^4,31^ have shown that higher WMH volumes are associated with an increased dementia risk, even over the span of a 14-year follow-up.^1^ Our study has also found an association of WMH volume with increased dementia risk over an average of 10 years in community-dwelling older adults with only limited cSVD burden (n = 1,738). Moreover, our study is the first to jointly assess whether baseline DAWM is also associated with increased long-term dementia risk in these individuals. However, no such association between DAWM and long-term dementia risk was found. This null result warranted further investigation by performing sensitivity analyses on individuals with a wider range of WMH burden in our study (n = 3,377 and n = 2,449). These analyses showed that properly accounting for the influence of baseline WMH on DAWM ratings requires a model interaction term between DAWM ratings and WMH volume. We have also developed an alternative approach consisting of calculating total abnormal white matter volume (by combining DAWM and WMH volumes), which showed a slightly increased hazard ratio to estimate dementia risk compared with a model with WMH volumes only. Quantifying DAWM volumes might therefore still prove useful in future studies on total white matter abnormalities in relation to dementia.

The lack of associations between baseline DAWM and cognitive decline and dementia risk is not likely a false negative due to low power: a sensitivity analysis (eAppendix 4) showed that our sample size is sufficient to provide a low chance of type II errors for the cognitive decline analysis in older adults with limited cSVD burden (n = 1,436). Instead, these null results might reflect various stages in the underlying pathophysiologic process of cSVD leading to cognitive decline and dementia. Relating early cSVD to cognitive outcomes 5 years later is complex. Within this time interval, both DAWM and WMH could increase, DAWM could possibly progress into WMH,^9^ or a part of the white matter changes could even regress.^7^ These processes can all happen at varying rates per individual, and the associated cognitive changes could lag behind this variation in white matter changes. Furthermore, the pathophysiologic changes underlying DAWM are likely also more minor compared with WMH. On postmortem imaging (n = 33), DAWM-like subtle T2-weighted signal changes showed less severe capillary endothelial damage, glial activation, and myelin attenuation on histology than found in WMH.^32^ Furthermore, our selection of community-dwelling older adults was likely to have less vascular risk factors, slower cSVD progression, and less cognitive decline compared with individuals with a more severe WMH burden.^6^ These older adults with DAWM but no severe WMH burden might not yet be at the tipping point for cSVD to start a pathophysiologic course toward dementia.

Strengths of this study include the use of a large, well-characterized longitudinal cohort, consisting of community-dwelling older adults that resulted in a strong external validity of our results. Because of the large amount of participants in our study, it is unlikely that our statistical analyses were underpowered for finding small effects related to DAWM. Another strength of our study is the comprehensive 5-year cognitive decline scoring as well as rigorous methodology and substantial interval times for long-term dementia outcomes.

Our study has several potential limitations. Follow-up data were not available for all participants, which could lead to an attrition bias toward healthier older adults with fewer vascular risk factors. Excluding participants with large infarcts (n = 26, 0.6% of sample) could also slightly contribute to a selection bias. However, selecting participants with limited baseline cSVD burden limits this bias because these individuals on average already have less vascular risk factors and healthier lifestyles.^9^ Another limitation could be that our cohort consists of a relatively ethnically homogeneous study population of Icelandic older adults,^12^ who, in general, have access to high-quality health care and educational systems. These factors can limit external generalizability of our results to countries with more diverse populations or more limited access to quality health care and education. Regarding dementia diagnoses, a limitation could be that diagnoses were determined by clinical consensus because the AGES study did not include biomarker measurements as recommended by the A/T/N framework.^33^ We were therefore not able to study the effect of DAWM in relation to different dementia diagnoses. A methodological limitation of our study was that we could not directly assess the evolution of DAWM into WMH in relation to cognitive change and dementia because this would have required developing a volumetric segmentation approach that is very labor-intensive to manually correct in a large cohort. Future studies could focus on this evolution to better understand the pathophysiologic course of white matter changes. Another limitation could be that visual DAWM rating was challenging because of the inherent difficulty of assessing diffuse intensity differences. However, to overcome this challenge, we maintained strict rating criteria and performed regular training and consensus meetings between raters to achieve an adequate interrater and intrarater agreement. A last limitation could be that the sensitivity to detect DAWM is lower on our 1.5T FLAIR MRI scans compared with current 3T MRI scans, as was suggested by a review on DAWM in patients with multiple sclerosis.^10^ This might have led to an underestimation of the statistical effects in our study.

In conclusion, in contrast to WMH, DAWM was not associated with baseline cognition, long-term cognitive decline, nor long-term dementia risk in community-dwelling older adults with limited cSVD burden. Although DAWM is associated with progression of cSVD, its role in the development of cognitive impairment and dementia remains unclear.

## Glossary

AGES: Age-Gene/Environment Susceptibility
BMI: body mass index
cSVD: cerebral small vessel disease
DAWM: “dirty-appearing” white matter
DSST: Digit Symbol Substitution Test
ePVS: enlarged perivascular spaces
FA: flip angle
FLAIR: fluid-attenuated inversion recovery
FOV: field of view
HR: hazard ratio
IRB: institutional review board
MNI: Montreal Neurological Institute
TE: echo time
TI: inversion time
TR: repetition time
WMH: white matter hyperintensity
